# High frame rate speckle tracking echocardiography to image the left ventricular mechanical activation sequence in healthy participants and patients with left bundle branch block

**DOI:** 10.1093/ehjimp/qyag086

**Published:** 2026-05-14

**Authors:** Konstantina Papangelopoulou, Laurine Wouters, Marta Orlowska, Annette Caenen, Gábor Vörös, Joris Ector, Jan D’hooge

**Affiliations:** Department of Cardiovascular Sciences, KU Leuven, Herestraat 49, Leuven 3000, Belgium; Department of Cardiovascular Sciences, KU Leuven, Herestraat 49, Leuven 3000, Belgium; Department of Cardiovascular Sciences, KU Leuven, Herestraat 49, Leuven 3000, Belgium; Department of Cardiovascular Sciences, KU Leuven, Herestraat 49, Leuven 3000, Belgium; Department of Electronics and Information Systems, Ghent University, Corneel Heymanslaan 10, Ghent 9000, Belgium; Department of Cardiovascular Sciences, KU Leuven, Herestraat 49, Leuven 3000, Belgium; Department of Cardiovascular Diseases, University Hospitals Leuven, University of Leuven, Herestraat 49, Leuven 3000, Belgium; Department of Cardiovascular Sciences, KU Leuven, Herestraat 49, Leuven 3000, Belgium; Department of Cardiovascular Diseases, University Hospitals Leuven, University of Leuven, Herestraat 49, Leuven 3000, Belgium; Department of Cardiovascular Sciences, KU Leuven, Herestraat 49, Leuven 3000, Belgium

**Keywords:** high frame rate echocardiography, strain rate, mechanical activation sequence, cardiac activation maps

## Abstract

**Aims:**

Electrical activation mapping is essential for identifying ablation targets in the treatment of cardiac arrhythmias. This study investigates high-frame-rate (HFR) speckle-tracking echocardiography (STE) as a non-invasive alternative to conventional electrophysiologic studies for constructing mechanical activation maps and defining left ventricular (LV) activation onset.

**Methods and results:**

Twenty healthy volunteers (HV) and 25 patients with a biventricular pacemaker (BiV) underwent HFR ultrasound scanning. Patients were scanned during BiV on and off, hence reintroducing native activation—left bundle branch block (LBBB) pattern. Five patients were additionally scanned after changing the LV lead pacing pole. A custom-made 2D-HFR-STE algorithm tracked the cardiac wall in the three conventional apical echocardiographic views. Strain rate (SR) curves were computed for each wall segment in a 16-segment LV model to measure the time between electrical and mechanical activation (i.e. time between QRS onset and first positive-to-negative zero-crossing in the SR curve). The timings were displayed in a bull’s-eye plot, representing each subject’s activation map. For most HV, activation started from mid-anteroseptum, at 23 ± 5 ms, spreading basal-inferolaterally at 50 ± 8 ms. During BiV off, the average septal activation was 36 ± 2 ms; the average lateral was 81 ± 21 ms (*P* < 0.01). During BiV on, the respective times were 53 ± 6 ms and 52 ± 6 ms (*P* = 0.3). Altering the LV lead pacing pole changed the lateral wall activation onset accordingly.

**Conclusion:**

HFR-STE was able to map normal activation, measure the known septal-to-lateral LBBB dyssynchrony, and identify the paced segment in 92% of the cases. HFR-STE could be a promising tool in constructing LV activation maps.

## Graphical Abstract

**Graphical Abstract:**
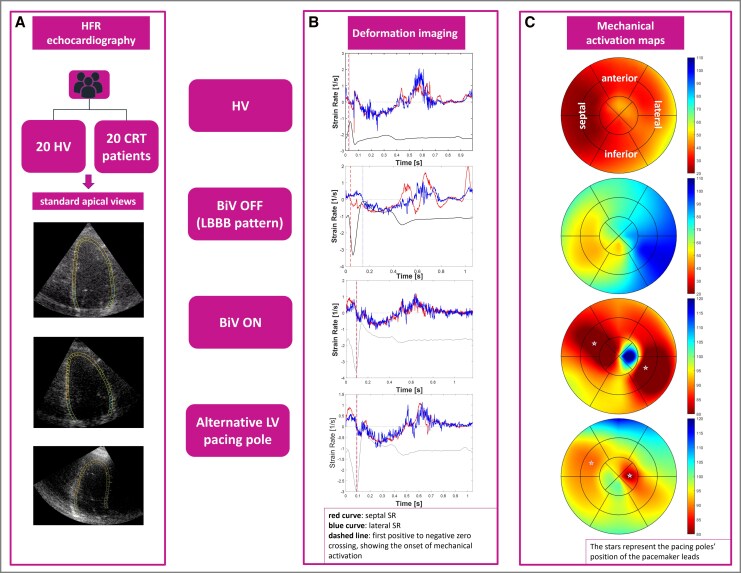
**(A)** All participants underwent HFR echocardiography (FR∼800fps). HFR echocardiographic views of the apical 4−, 2− and 3− chamber view (from top to bottom) in a representative healthy volunteer (HV), showing the manually placed contour, subdivided into six segments for each view. **(B)** Strain rate (SR) examples from the mid-septal (red - dashed line) and mid-lateral (blue - dotted line) segment from the three different groups: HV, heart failure patient with LBBB (BiV off), during biventricular pacing (BiV on), and after changing the pacing pole of the LV lead. The vertical lines show the position of the first positive to negative zero crossing on the SR curve after the onset of QRS (i.e. onset of segmental mechanical activation) for two segments. Time 0 is set as the QRS onset. **(C)** The activation timings (in ms) extracted from all segments were then placed in a 16-segment bull’s eye plot. From top to bottom: a representative activation map from an HV and an HF patient (BiV off, BiV on, and paced from an alternative pole of the LV lead). The stars represent the pacing poles position of the pacemaker leads. The representation of the myocardial walls on the bull’s eye are shown on the example from the HV **(C)**; the septal and lateral walls are further divided into antero- and infero-septal and lateral, respectively. From outer to inner, the circles represent basal, mid, and apical segments.

## Introduction

Cardiac arrhythmias represent a major cause of worldwide morbidity and mortality.^1,2^ The accurate localization of the arrhythmia substrate, i.e. the site of the electrical abnormal activation, whether in the form of an ectopic focus or a re-entrant circuit, is essential in their effective treatment.^2,3^

Several diagnostic tools are currently being used in order to map the cardiac electrical activation, with the gold standard being an invasive electrophysiological study (EPS) (with or without electroanatomic mapping). During this procedure, catheters, inserted into the heart, sense electrical signals along the cardiac wall, thereby identifying regions of abnormal activation.^4^ However, this invasive technique carries increased risk, such as bleeding and other potentially life-threatening complications.^5^ Besides, the clinical arrhythmia cannot always be induced during an EPS,^6,7^ rendering the study non-diagnostic.

A promising and non-invasive alternative to EPS is high-frame-rate (HFR) echocardiography, enabling ultrasound image reconstruction at 500–1000 fps,^8^ compared with the conventional frame rate of 50–80 fps. Echocardiography, being a low cost, safe, and readily available imaging modality, plays a central role in the diagnosis and management of patients with suspected cardiac pathologies.

This high temporal resolution allows to resolve short-lived cardiac events, such as the onset of myocardial shortening, yielding insight into the mechanical activation. Several studies have shown the value of combining HFR echocardiography and myocardial deformation imaging to construct mechanical activation maps, based on a novel technique called clutter filter wave imaging,^9^ or on one-dimensional myocardial motion estimation.^10–12^

As a user-friendly and angle-independent alternative, our group developed an HFR speckle-tracking (STE) methodology.^13^ Speckle-tracking analyses the cardiac tissue motion in 2D by using the pattern of speckles in B-mode images naturally resulting from the ultrasound beam reflection in the myocardium. The performance of our STE extension on HFR images has been validated in healthy volunteers undergoing an exercise stress echocardiogram.^14^

Our study is a proof-of-concept study testing the feasibility of STE on HFR B-mode images in order to create cardiac mechanical activation maps of the left ventricle. Therefore, we applied the proposed methodology to participants with known left ventricular activation patterns. We included healthy volunteers who have, by definition, a normal left ventricular activation, already described in the literature^15^ as well as patients with heart failure (HF) and a left bundle branch block (LBBB) pattern of activation, treated with Cardiac Resynchronization Therapy (CRT).

LBBB is an abnormality of the cardiac conduction system. Briefly, in such cases the left bundle branch, which is normally responsible for the fast propagation of the electrical signal to the lateral wall of the LV, is no longer functional.^16^ This results in an early septal activation (activated from the still functional right bundle branch-RBB), followed by propagation of the electrical signal via the slow conducting myocardial cells towards the lateral wall, resulting in a delayed, late activation of the latter. Patients with HF, reduced ejection fraction (LVEF), and such a conduction abnormality are eligible for the so-called cardiac resynchronization therapy (CRT).^17^ In this case, a pacemaker with biventricular leads is implanted, with one lead pacing the septal area within the right ventricle and the other pacing the LV lateral wall (through the coronary sinus). The LV lead is normally quadripolar, and depending on patient-related factors, any of the four poles can be used for pacing.

LBBB has a known and well-described pattern of a dyssynchronous activation (early septal-late lateral wall), with significant timing differences between the activation of two LV myocardial walls. These pronounced delays make it easier to detect differences in activation timing and facilitate the initial goal of the study, which was to investigate whether HFR-STE can accurately capture temporal differences during LV mechanical activation.

In addition, and given the fact that the pacing sites during biventricular pacing can be localized from fluoroscopy, we were also able to test the spatial accuracy of our proposed methodology.

By applying our HFR-STE on participants with known LV activation sequences, this study aimed to validate the feasibility of our proposed methodology in mapping the LV mechanical activation sequence.

## Methods

### Study population

This study was prospectively conducted in the University Hospitals Gasthuisberg, Leuven, Belgium. A total of 20 healthy volunteers (HV) and 25 HF patients treated with CRT were included. The number of participants was *ad hoc* selected, based on previous experience, which suggests that this relatively small number of volunteers and patients would be sufficient to draw meaningful conclusions. HV were defined as subjects with no known cardiac pathology and no pathological findings from the echocardiogram and 12-lead ECG. Patients’ inclusion criteria were LBBB conduction abnormality before CRT implantation and a percentage of biventricular pacing (BiV) of at least 95%. The latter means that at least 95% of the ventricular complexes were induced by BiV pacing and only (or less than) 5% is due to intrinsic activation, which indicates successful resynchronization, as per the HF guidelines.^17^ In order to minimize CRT-induced remodelling, we only included patients with recent (within 3 months) pacemaker implantation. Participants with poor acoustic windows, atrial fibrillation and 2nd or 3rd degree atrioventricular block were excluded from the study. All patients had undergone cardiac magnetic resonance (CMR) prior to CRT implantation, as part of the clinical routine. The study was approved by the Ethics Committee of the University of Leuven (S66094) and all participants gave written informed consent.

### Study design

Age, sex, height, weight, and blood pressure were recorded for all participants. A 12-lead ECG was also acquired. For the patients, ECG data were acquired both during biventricular pacing on and off (BiV on and BiV off, respectively).

Echocardiographic data were acquired from the patients first during BiV (DDD mode), but also after switching the BiV off (AAI mode), in order to reintroduce the LBBB pattern of intrinsic activation. Shortly, in DDD, the pacemaker senses intrinsic activation from both atria and ventricles and can also pace both. In the case of CRT, this function optimizes BiV pacing, maximizing the synchronicity of ventricular contraction. During AAI, only the atria can be ‘sensed’ and paced. As a result, all ventricular activity will be induced by intrinsic electrical activation. The pacing pole positions were determined from fluoroscopic images during CRT pacemaker implantation. All patients had a quadripolar LV lead. For five patients, data were also acquired after temporarily modifying the pacing pole of the left ventricular (LV) electrode. As an alternative pacing pole, we opted for the one that did not cause diaphragmatic stimulation and that resulted in electrical capture, as confirmed by the ventricular electrogram during the CRT interrogation. Supplementary data online, *Table S1* shows the original and the alternative pacing pole for all five patients. The settings were set back to the original ones after data acquisition.

Finally, to evaluate the reproducibility of our measurements, a second dataset from five of the HV was acquired, within 1 month of the first one, taking care that the individual heart rate (HR) and loading conditions were not altered.

### Echocardiography

#### Conventional echocardiography

All participants underwent a standard echocardiographic examination using a GE E95 ultrasound scanner (GE Vingmed, Horten, Norway) and all measurements were performed according to current guidelines.^18^

#### HFR echocardiography

HFR echocardiography was performed with a GE Vivid E95 ultrasound scanner, equipped with an M5Sc-D phased array transducer (GE Vingmed, Horten, Norway), with a central frequency of 3.5 MHz. The three standard apical views (apical 4−, 2−, and 3-chamber view) were acquired at 827 ± 92fps (*Graphical abstract A*) using five diverging waves and an imaging depth of 14–15 cm, ensuring full LV coverage. During the echocardiographic acquisitions, one lead of the surface ECG was simultaneously recorded. All measurements were performed in three cardiac cycles and values were subsequently averaged.

#### Deformation imaging

For initialization of LV wall segmentation, reference points were manually placed at end-diastole, at the border of endo- to mid-LV myocardium, to avoid bias in the motion estimation due to the strong reflecting pericardium. Then, a contour was created by fitting a spline to the reference points, and tracked throughout the cardiac cycle by an in-house developed 2D HFR-STE algorithm, based on cross-correlation, in Matlab R2021b (Mathworks, Natick, MA, USA).^13^ The LV was divided into a standard 16-segment model.^19^ Each segment was sub-segmented into two, sub-segmental longitudinal strain curves were calculated, and the average of each pair was used (i.e. a max of 32 curves was used for each subject to create a 16-segment bull’s eye plot), following the Lagrangian strain definition.^19^ Strain rate (SR) curves were computed as the time derivative of the calculated strain. Additional technical details are provided in the Supplementary data, as well as in our previous work.^13,14^

#### Activation maps

The temporal distance (in ms) between the electrical and the mechanical onset of activation was calculated for each LV segment. The electrical activation onset was defined as the beginning of the QRS complex in the lead recorded during the echocardiogram, whereas the mechanical activation onset was defined as the start of segmental shortening, i.e. the first positive to negative zero-crossing in the local SR curve (*Graphical abstract B*). The first zero-crossing after QRS onset was automatically calculated, and manually adjusted if needed, to ensure it was followed by a significant trough of at least 10 ms duration, corresponding to myocardial shortening occurring during isovolumic contraction (IVC). The 10 ms threshold was selected *a priori* based on the assumption that it would be long enough to represent a physiologically meaningful event, whereas remaining short enough to ensure a more robust detection of the zero-crossings, given the increased noise of the HFR data.

The extracted timings were used to create a mechanical activation map, with each segment’s timing placed at the segment’s centre in a bull’s-eye plot. To ensure a smooth appearance in the bull’s eye plot, the values between the estimated timings were interpolated (*Graphical abstract C*).

It should be noted that as in previous studies, ‘onset of mechanical activation’ was defined as the onset of myocardial shortening, which does not account for the complex electromechanical processes taking place between the electrical depolarization and mechanical contraction.^20^ In the current study, we are using the term mechanical activation to refer to the onset of myocardial shortening, while acknowledging that, even though there is a strong correlation between the two,^21^ this is not a direct measure of electrical activation nor the true onset of mechanical activation, which indeed may start with an isometric phase. This is thus not accounted for in our methodology. We have chosen to keep the nomenclature ‘onset of mechanical activation’ to remain consistent with previous studies.^11,21,22^

The duration of LV mechanical activation was estimated as the difference in activation time between the earliest and latest activated myocardial segment. The duration of ventricular electrical activation was defined as the QRS duration.

In order to estimate intra- and inter- observer variability, the SR curves derived from the three apical views of five participants (four HV and one HF patient—both during BiV on and BiV off) were re-analysed by KP and LW.

### Localization of the pacing pole

Based on the fluoroscopic images acquired during CRT implantation, the position of the pacing pole for both right ventricular (RV) and LV lead was marked. The right anterior oblique view identified the pole’s location in the basal, mid, and apical segments, while the left anterior oblique view determined its position in the antero-, mid-, and infero-lateral segments.^23^ In order to localize the RV lead pacing pole, the anatomic position of the RV relative to the LV was considered. Finally, the absolute difference (in segments) between the fluoroscopically and echocardiographically measured earliest activated segment was calculated on the bull’s eye plots, for both RV and LV electrodes. Supplementary data online, *Figure S1* shows two characteristic examples of the bull’s eye plots during BiV and how this difference was calculated.

### Statistical analysis

Continuous variables are expressed as mean ± SD and categorical variables as percentages and frequencies. Normality of data was assessed with the Shapiro–Wilk test. Comparisons between groups were made using one-way analysis of variance (ANOVA) with Bonferroni correction or Kruskal–Wallis test with Dunn post-hoc analysis, as appropriate. Correlation between electrical and mechanical activation was calculated using Spearman’s Rank test. Reproducibility of mechanical activation maps was tested using Bland Altman analysis, while the intraclass correlation coefficient (ICC) was also estimated (two-way mixed model, absolute agreement). The latter were estimated for test–retest reproducibility, as well as for intra- and inter-observer variability. Statistical significance for all tests was set at a 0.05 level. All statistical analyses were performed with SPSS Statistics Version 29.0 (IBM, Armonk, NY).

## Results

Five patients were excluded from the study: one because of poor HFR image quality, two because, after switching the CRT off, the 12-lead surface ECG no longer showed a LBBB pattern of activation, and two because of pacemaker dependency. As a result, 20 HVs and 20 patients were enrolled in this study. In addition, we excluded myocardial segments with significant ischaemia and scar burden, as defined by a ≥50% transmural late gadolinium enhancement in CMR, in order to avoid adding segments not actively contracting, but rather tethered by nearby activated myocardium.

### Demographic characteristics

*Table 1* shows the baseline characteristics of all participants. The mean age of the HV was 29.8 ± 5.1 years and 11/20 (55%) were male, whereas the mean age of the HF patients was 66.4 ± 10.3 years and 14/20 (61%) were male. Four of the patients had ischaemic cardiomyopathy, whereas the rest had dilated cardiomyopathy of non-ischaemic origin.

**Table 1:** Baseline characteristics of study participants

	HV (*n* = 20)	BiV off (*n* = 20)	BiV on (*n* = 20)	*P*-value
***Clinical***				
Age (y)	29.8 ± 5.1	66.4 ± 10.3		**< 0.001**
Male	11 (55%)	13 (65%)		0.748
SBP (mmHg)	N/A	136.3 ± 20.2		N/A
DBP (mmHg)	N/A	78.05 ± 13.9		N.A
HR (beats/min)	64.2 ± 9.2	63.5 ± 10	64.2 ± 9	0.893
QRS duration (ms)	85.5 ± 9.3	146.5 ± 20.1^§^	139.6 ± 17.5^§^	**< 0.001**
***Echocardiographic***				
LVEDD (cm)	4.6 ± 0.4	5.7 ± 1.3^§^	5.6 ± 1.3^§^	**0**.**007**
IVSd (cm)	0.8 ± 0.1	0.9 ± 0.2	0.9 ± 0.3	0.081
PWd (cm)	0.8 ± 0.1	1 ± 0.2	0.9 ± 0.2	0.063
EDV (ml)	97.7 ± 23.4	146.3 ± 83.3^§^	144.8 ± 77.6^§^	**0**.**048**
ESV (ml)	34 ± 9.9	85.6 ± 73.3^§^	82 ± 71.3^§^	**0**.**016**
LVEF (%)	64.8 ± 6.6	47 ± 13.1^§^	48.9 ± 13.3^§^	**< 0.001**
LAVI (mL/m^2^)	22.9 ± 0.4	34.1 ± 12.9^§^	36 ± 12.1^§^	**0**.**004**
E (m/sec)	0.81 ± 0.14	0.59 ± 0.29^§^	0.63 ± 0.31^§^	**0**.**047**
Transmitral E/A	1.73 ± 0.42	0.9 ± 0.64^§^	1.19 ± 1.19^§^	**0**.**039**
DT (ms)	192 ± 35.4	253.7 ± 84.9^§^	245.9 ± 78.6^§^	**0**.**044**
e’ septal (m/s)	0.14 ± 0.02	0.05 ± 0.01^§^	0.05 ± 0.01^§^	**< 0.001**
e’ lateral (m/s)	0.19 ± 0.02	0.06 ± 0.02^§^	0.06 ± 0.02^§^	**< 0.001**
E/e’−average* ratio	5 ± 0.8	11.9 ± 5.3^§^	11.6 ± 6.3^§^	**< 0.001**
GLS (%)	19 ± 1.9	11.5 ± 3.4^§^	10.7 ± 3.4^§^	**< 0.001**
Mech. activation dur. (ms)	27.1 ± 7	68.8 ± 24.2^§^	34.7 ± 13.4^§†^	**< 0.001**

Continuous variables are expressed as mean ± SD; categorical variables as absolute number (percentages). HR: heart rate, LVEDD: left ventricular end-diastolic diameter, IVSd and PWd: end-diastolic thickness of interventricular septum and inferolateral wall, respectively, EDV and ESV: end-diastolic and end-systolic volume, respectively, LVEF: left ventricular ejection fraction, LAVI: left atrial volume indexed (per body surface area-BSA), DT: deceleration time, GLS: Global longitudinal strain, Mech. activation dur.: duration of LV mechanical activation

Significance level was set at *P* ≤ 0.05 and is higihlighted in bold. Significance for between-groups differences: §*P* ≤ 0.05 vs. HV; †*P* ≤ 0.05 vs. BiV off.

### Activation maps

**Figure 1:**
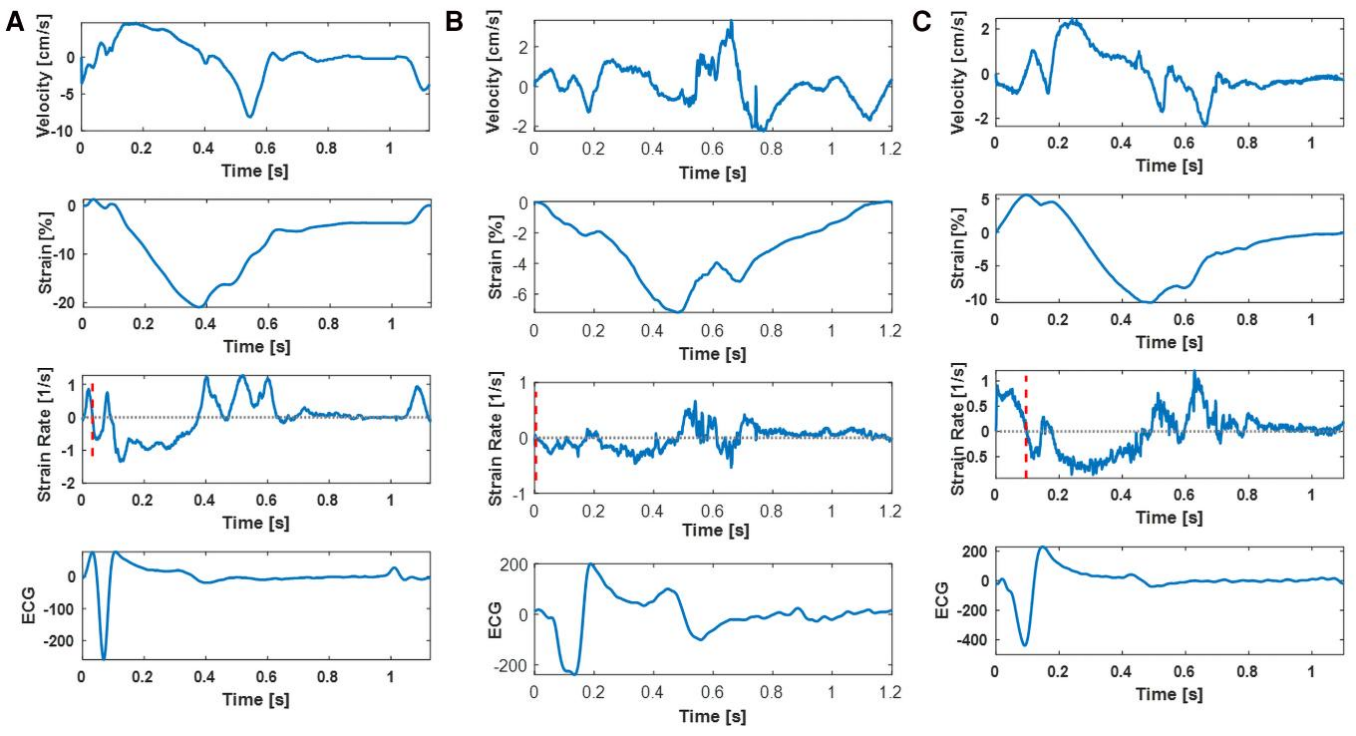
From top to bottom: segmental velocity, strain, SR, and ECG. These examples were taken from the mid-septal segment of an HV (*A*) and an HF patient during BiV off (*B*) and BiV on (*C*). The vertical dashed lines on the SR curves represent the beginning of systole (onset of mechanical activation). Abbreviations as in the *Graphical abstract*.

**Figure 2:**
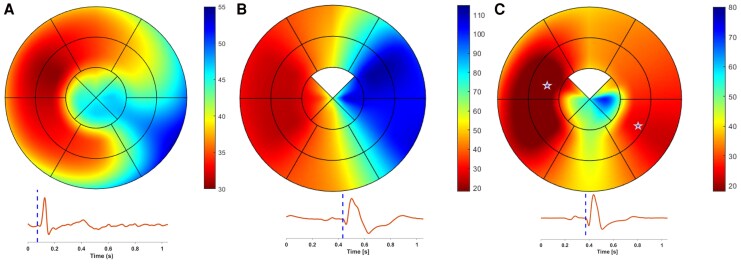
Activation map in ms from a HV (27y, M) **(***A***)** and a HF patient (51, M) during BiV off (*B*) and BiV on (*C*), along with their respective one ECG lead recording. The stars in C represent the pacing poles' position of the two pacemaker leads. The apical anterior segment from the patient was not tracked due to low image quality. The color scale for the three bull’s eye plots represents time in ms and is such that highlights the intrasubject variability. Abbreviations as in the *Graphical abstract*. The corresponding 12-lead ECGs are shown in Supplementary Data, in Supplementary data online, *Figure S8*.

Tracking was feasible in 90% of the segments where strain and SR curves showed a physiological pattern (*Figure 1*).^24^ The detailed segmental onset and end of LV mechanical activation for the three study groups are shown in *Table 2*, and characteristic examples of each group are shown in *Figure 2*. Supplementary data online, *Figure S2* shows examples of SR curves where the onset of mechanical activation needed to be manually adjusted.

**Table 2:** Onset and end of LV mechanical activation for the three study groups

Myocardial segments	HV		BiV off		BiV on	
	onset	end	onset	End	onset	End
Basal anterior						1 (5%)
Basal anteroseptal	1 (5%)		1 (5%)			1 (5%)
Basal inferoseptal		1 (5%)	1 (5%)		4 (20%)	1 (5%)
Basal inferior						1 (5%)
Basal inferolateral		19 (95%)		7 (35%)	1 (5%)	2 (10%)
Basal anterolateral				5 (25%)	1 (5%)	1 (5%)
Mid anterior						
Mid anteroseptal	14 (70%)		9 (45%)		5 (25%)	
Mid inferoseptal	5 (25%)		7 (35%)		3 (15%)	
Mid inferior						
Mid inferolateral				2 (10%)	3 (15%)	
Mid anterolateral				6 (30%)	1 (5%)	
Apical anterior						5 (25%)
Apical septal			2 (10%)		1 (5%)	
Apical inferior						5 (25%)
Apical lateral					1 (5%)	1 (5%)

For 95% of the HV, LV mechanical activation started at the mid-anteroseptal segment, the activation wave quickly propagated towards the mid-ventricular and apical segments, followed by the basal segments, and ended at the basal-inferolateral segment (*Figure 2A*). On average, mechanical activation started 23.1 ± 5.1 ms and ended 50.1 ± 7.9 ms after QRS onset, with a total duration of 27.1 ± 6.9 ms.

During the LBBB pattern, LV activation started at the septal wall, and then slowly propagated through the anterior and inferior walls towards a late lateral activation (*Figure 2B*). Mechanical activation started on average 28.1 ± 9.2 ms and ended 96.9 ± 25 ms after QRS onset, whereas its total duration was 68.8 ± 24.2 ms.

During BiV on, two sites of activation onset were recorded, one at the septal and one at the lateral wall of the LV (*Figure 2C*). The activation wave showed a greater variability compared with either native conduction pattern, depending on the pacemaker lead position and the programming settings. Mechanical activation started on average 28 ± 9 ms and finished 77 ± 25 ms after QRS onset, whereas the total duration was 35 ± 13 ms.

The detailed activation pattern for each participant is described in Supplementary data online, *Table S2*. The bull’s eye plots representing the average mechanical activation sequence for the three groups are shown in Supplementary data online, *Figure S3*.

### Comparison of activation times between septal and lateral wall

**Figure 3:**
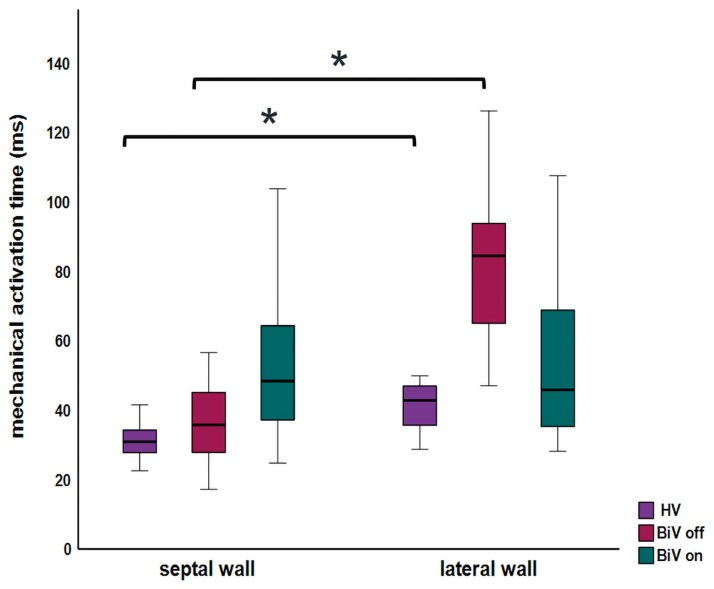
Clustered boxplot showing the average activation time of the septal and the lateral wall for the three subject categories (* *P* < 0.001). Abbreviations as in the *Graphical abstract*.

There was no significant difference between the average activation times of the septal and lateral wall for the BiV on group (53 ± 6 vs. 52 ± 6 ms). In contrast, the BiV off group exhibited a significant difference (36 ± 2 vs. 82 ± 21 ms; *P* < 0.001 in *Figure 3*), showing the dyssynchronous activation of the septal and lateral walls, i.e. the well-known LBBB pattern. Despite a shorter total activation time than the LBBB pattern, the HV also showed a significant difference in septal and lateral wall activation (31 ± 5 vs. 42 ± 7 ms; *P* < 0.001), demonstrating the gradual propagation of myocardial activation from the septum to the lateral wall. Supplementary data online, *Figure S4* shows the relationship between the activation timings of the septal and lateral wall of the different groups.

### Comparison between duration of electrical and mechanical activation

**Figure 4:**
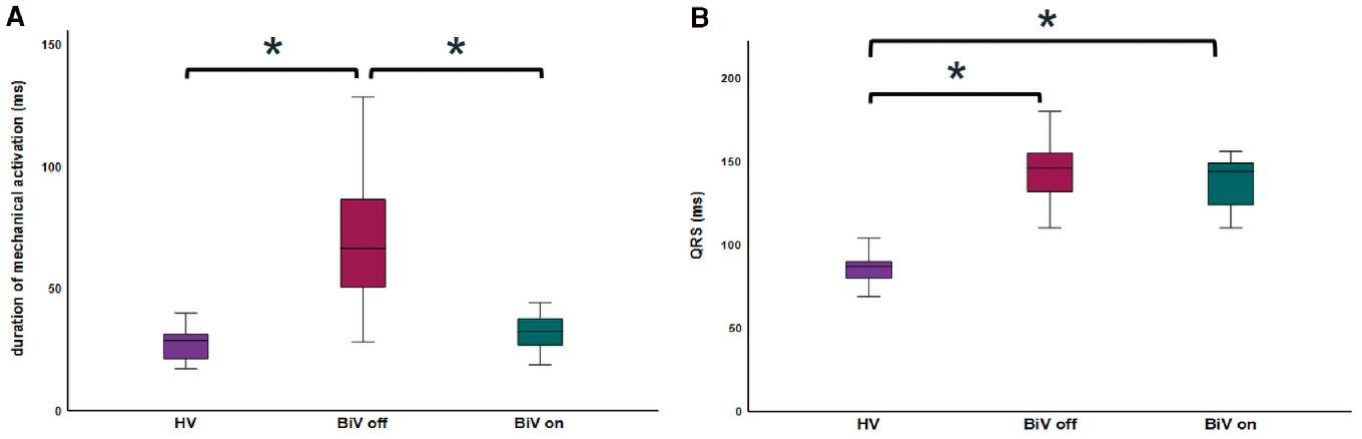
Boxplots showing the duration of mechanical (*A*) and electrical (*B*) activation for the three study groups (*P < 0.001). Abbreviations as in the *Graphical abstract*.

Comparing the duration of mechanical activation between the three groups showed a statistically significant difference between the BiV off group and the two other groups (*P* < 0.001), but not between the HV and the BiV on (*P* = 0.490) (*Figure 4A*). In contrast, the duration of electrical activation differed between the HV and the HF patients, both during BiV on and off, but not between the patients’ native rhythm and the resynchronized activation pattern (*Figure 4B*).

The correlation between electrical and mechanical activation is discussed in the Supplementary Data.

### Accuracy in identifying segmental activation onset

The difference in localizing the activation onset between our method and fluoroscopic images is tabulated in *Table 3*, showing an accuracy of 0–1 segments for most subjects (88% for RV lead and 96% for LV lead).

**Table 3:** Difference (in myocardial segments) between the expected earliest activated segment and the measured earliest activated segment in all patients (*n* = 25; including the five patients in which the LV lead pacing pole was modified)

CRT pacemaker lead	Segmental difference		
	0	1	2
RV lead	14 (56%)	8 (32%)	3 (12%)
LV lead	15 (60%)	9 (36%)	1 (4%)

**Figure 5:**
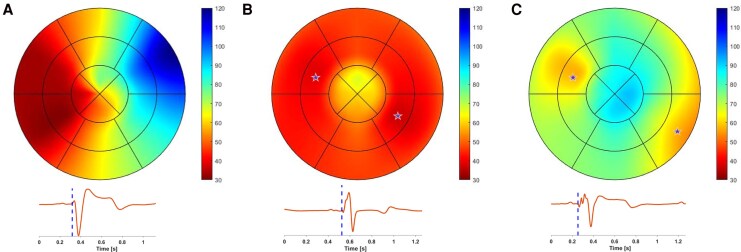
Activation map in ms from one HF patient (67y, M) during BiV off (*A*) and during BiV on with the electrode tip (*B*) or the electrode ring (*C*) as the pacing pole of the LV lead, along with their respective one ECG lead recording. The stars represent the pacing Poles position of the two leads. Abbreviations as in the *Graphical abstract*.

For the five patients whose LV pacing pole was modified, the earliest activated myocardial segment of the lateral wall changed in accordance with the position of the new pacing pole in all patients. A characteristic example of the different activation maps is shown in *Figure 5*.

### Reproducibility

Among the five re-scanned HV, there was no statistical difference between scans for any segment (see Supplementary data online, *Table S3*). The ICC was 0.846 (95% CI 0.759–0.902, *P* < 0.001), demonstrating good reproducibility across echocardiographic examinations. The Bland–Altman plot (see Supplementary data online, *Figure S5*) showed a mean difference of −0.1 ms and 95% limits of agreement between −13.9 and 13.7 ms. Supplementary data online, *Table S4* shows the different echocardiographic measurements of the repeated examinations.

Both the intra- and inter- rater reliability were good, with an ICC of 0.89 (95% CI 0.856–0.917 *P* < 0.001) and 0.848 (95% CI 0.801–0.884, *P* < 0.001), respectively. The respective Bland–Altman plots are shown in Supplementary data online, *Figure S6*.

## Discussion

In this initial proof-of-concept study, the feasibility of HFR-STE, a novel non-invasive method, in mapping the LV mechanical activation sequence was tested. By using the onset of myocardial shortening as a surrogate for the onset of mechanical activation, our method was able to reproduce the previously described activation sequence in HV and CRT-treated patients with LBBB. This confirms that HFR-STE can be employed in mapping the LV mechanical activation sequence.

### Mechanical activation pattern

For the HVs, the mid-septum was generally the first to be activated (95% of cases). The activation wave propagated towards the apical and basal segments, the anterior and inferior walls, and ended at the basal-inferolateral segment. During native conduction in LBBB patients, the earliest and the latest activated segment varied among the different septal and LV free wall segments, respectively, probably depending on the anatomical site of the block. During BiV on, two sites of activation onset were recognized, one in the septal and one in the lateral wall, both in proximity to the respective pole of the two pacing electrodes.

As previously mentioned, we opted for patients with recently implanted CRT in order to avoid potential (electrical and/or mechanical) remodelling. It has been shown that long-term CRT therapy can induce both electrical,^25^ as well as mechanical^26^ remodelling. We thus ensured that native conduction still had an LBBB pattern of activation.

These findings are in accordance with previous studies in healthy volunteers,^15,27^ LBBB patients with BiV off^28–30^ and LBBB patients with BiV on,^28,31^ utilizing both invasive and non-invasive techniques.

### Activation timings

Normal mechanical activation duration was significantly shorter than that of the LBBB pattern, which in turn decreased after resynchronization, i.e. during BiV on. Moreover, there was a significant difference between the activation timings of the septal and lateral wall during BiV off, which further highlights the known dyssynchrony in myocardial wall contraction that characterizes the activation pattern of LBBB.

The activation timings between the septal and lateral wall also differed significantly in the HV. However, the absolute difference was considerably smaller compared with the LBBB one and, the total duration of mechanical activation was significantly shorter compared with the BiV off state. This is consistent with the sequential fast propagation of the normal activation wavefront, in contrast with the slow and dyssynchronous activation pattern observed during LBBB.

Notably, the average septal activation during BiV on was greater than that during BiV off. During BiV off, the septum is intrinsically activated by the RBB, branching into the fast conducting Purkinje fibres, resulting in a fast propagation of the activation front throughout the septum. During BiV on the septum is activated from the pacing lead, placed on the RV septal side, implying that the activation is likely not propagated through the Purkinje fibres, but rather through the slow conducting myocardial cells. We assume that this is possibly the reason for the discrepancy in the septal activation time during BiV off vs. BiV on.

### HFR echocardiography to identify activation onset

In most patients in this study, HFR echocardiography accurately identified the earliest activated myocardial segment when compared to fluoroscopy during pacemaker implantation. Additionally, a repositioning of the pacing pole of the LV lead was correctly identified by HFR-STE in all five patients, implying that HFR-STE can detect subtle changes in the onset of activation. Furthermore, the ICC analysis demonstrated good reproducibility of HFR-STE in constructing mechanical activation maps in five HV, as well as good intra- and inter- variability.

Different medical imaging modalities have been used for LV activation mapping, such as MRI,^29^ as well as for CRT optimization, such as conventional STE.^32^ Various methods have been used in an attempt to detect patients that would most likely benefit from CRT; Tissue Doppler Imaging (TDI),^33^ MRI tagging,^34^ Electrocardiographic Imaging (ECGi),^35^ showing promising results. Each technique, however, comes with its own limitations. ECGi is time-consuming and fairly complicated, requiring dedicated personnel, preventing its integration into clinical practice.^36^ MRI and conventional STE lack temporal resolution^37^ for accurately defining short-lived cardiac events, e.g. segmental activation onset. Conversely, Tissue Doppler Imaging (TDI) offers a higher temporal resolution, however its applicability is limited due to its angle dependency and one-dimensional estimation of myocardial motion.^38^

Due to the aforementioned limitations, current guidelines still use QRS morphology and duration criteria to identify appropriate CRT candidates.^17^ However, a large number of patients seem not to benefit from CRT, despite meeting the CRT criteria,^39^ whereas different biomarkers had proven beneficial when added to the ‘standard’ QRS criteria.^40^ The combination of high temporal resolution with the user-friendly technique of STE facilitates the use of non-invasive modalities in cardiac activation mapping, defining not only the onset but also the sequence of LV mechanical activation, and could thus provide possible additional information for CRT optimization.

Several studies have already shown the feasibility of using HFR imaging to identify activation onset,^9,31^ and have applied their technique in the same patients group^22^ with promising results. The technique utilized in the current study represents an extension of the HFR echocardiographic technique used by these previous studies: it estimates motion in 2D (both parallel and perpendicular to the ultrasound beam) instead of 1D (along the ultrasound beam). As such, it facilitates data processing as segmental tracking in the 2D image is done automatically and could provide more information on the complex cardiac motion. In addition, STE has already been implemented in clinical practice, with most sonographers familiar with its use, implying the easier implementation of our technique in the clinical workflow.

Our study showed that HFR-STE can reliably construct LV mechanical activation maps and measure LV mechanical activation time. HFR echocardiography could also improve patient selection for CRT, currently relying on ECG criteria alone, benefiting just 2/3 of patients.^41^ A recent study^41^ has shown that HFR echocardiography distinguished ‘super-responders’ (patients with a ΔLVEF ≥ 20%) from non-responders (ΔLVEF ≤ 5%), but could not distinguish ‘responders’ (10%≤ΔLVEF < 20%) from ‘non-responders’. Additionally, previous studies have shown that strain and SR can offer valuable insight on both contractility,^42^ as well as stiffness,^43^ which could thus prove useful in improving CRT patient selection. However, the scope of our study was to assess whether our proposed technique (i.e. HFR-STE) can measure local activation timings, rather than investigate the possible value of novel biomarkers in improving CRT patient selection. Further studies, which should also include a more detailed analysis of deformation imaging curves, e.g. different strain patterns, are needed to assess its potential role in optimizing CRT patient selection.

HFR-STE also correctly identified the earliest activated LV myocardial segment, often the ablation target for cardiac arrhythmia treatment. The additional information provided by our proposed non-invasive technique could decrease the time needed for accurate localization of the ablation target during EPS, as well as the time of the ablation itself, minimizing the patient-related risk. However, in order to verify this hypothesis, further studies should be performed on cardiac arrhythmia patients with (clinically indicated) electroanatomical mapping as a gold standard reference methodology.

As previously mentioned, we defined the onset of mechanical activation as the onset of myocardial shortening, consistent with prior literature.^11,21,22^ However, we acknowledge that the onset of myocardial shortening might not always reflect the true onset of mechanical activation, especially in cases of cardiac pathology, such as in LBBB.^44^ Besides, and although segments with significant scar were excluded, underlying myocardial disease may still influence the timing of myocardial shortening, through changes in contractility and regional mechanical properties. However, while the onset of mechanical activation cannot be directly measured, particularly in a clinical setting, the onset of myocardial shortening can. Although the onset of local shortening is impacted by local electromechanical coupling and local loading conditions,^45^ it seems an acceptable first proxy to assess the onset of local mechanical activation. As electromechanical coupling and loading can vary throughout the LV, however, (particularly during disease, e.g. LBBB), a spatially variant discrepancy between the onset of local shortening and the onset of mechanical activation could arise.

Despite these limitations, measuring the onset of myocardial shortening remains a reproducible and clinically feasible biomarker, which allows for comparison with prior studies. However, we acknowledge the complex electromechanical interplay of the myocardium and we believe that future studies combining deformation imaging -derived biomarkers with direct measures of electrical activation are needed to further clarify the relationship between mechanical shortening and true mechanical activation.

### Study limitations

Our study has several limitations. First, although age influences cardiac conduction, our HVs were not age-matched with the HF patients. Differences in LV size and function between the two study populations may have affected the observed differences. Although following up HF patients in time allows for investigating possible differences in the mechanical activation timings between CRT ‘responders’ and ‘non-responders’, it fell outside the scope of this feasibility study. Furthermore, our methodology uses a standard 16-segment model for the LV, which may have overlooked detailed information of the LV activation sequence, and experiences relatively low tracking feasibility of the apical segments (probably due to the low signal-to-noise ratio of HFR imaging). In addition, we constructed mechanical activation maps by using only the three standard apical views. This limits the spatial resolution of our technique to the size of a myocardial segment (∼20 mm), and future work should investigate whether sub-segmentation of the LV (in combination with 3D imaging) would allow us to increase its spatial resolution for more accurate localization of arrhythmia onset. Since our contour was placed on the inner part of the left ventricular walls, only the endocardial motion was tracked. We thus were not able to measure the transmural activation time, nor were we able to measure the later epicardial activation timings.^46,47^ The latter could account for the rather short total mechanical activation, compared to already described activation timings. Finally, since electroanatomical mapping was not performed in our study population due to the lack of clinical indication, we based the onset of electrical activation on fluoroscopic images. The latter provides anatomical information of lead placement that might not necessarily reflect true electrical activation.

### Conclusion—future perspectives

In this study, we investigated the feasibility of applying a non-invasive, novel imaging technique as a diagnostic tool for cardiac mechanical activation sequence. We applied HFR-STE on subjects with a known, already described in the literature, pattern of LV mechanical activation sequence. Furthermore, we showed that this technique can correctly localize the onset of mechanical activation in CRT patients based on pacing pole position as seen in fluoroscopy images. Further studies are warranted to investigate whether these preliminary findings can be translated and applied in arrhythmia patients with a more complicated activation pattern. Additionally, a head-to-head comparison with invasive data (electroanatomical mapping) is needed to verify our findings in an arrhythmia population study. We are currently developing the translation of our HFR-STE to 3D HFR images, without the need for LV geometry assumptions or interpolation between 2D echocardiographic images.

## Supplementary Material

qyag086_Supplementary_Data

## Data Availability

Data can be made available upon reasonable request.

## Lead author biography

Dr. Konstantina Papangelopoulou is a trained cardiologist currently pursuing a Doctorate in Biomedical Sciences at KU Leuven, Belgium. She holds the General Cardiology Certification from the European Society of Cardiology and the Adult Transthoracic Echocardiography from the European Association of Cardiovascular Imaging, whereas she also completed the Postgraduate Course in Advance Medical Imaging at KU Leuven, Belgium. Her research interests include multimodality cardiac imaging, including cardiac magnetic resonance and echocardiography, particularly novel techniques such as high frame rate echocardiography.
