# Supplementary material for: High frame rate speckle tracking echocardiography to image the left ventricular mechanical activation sequence in healthy participants and patients with left bundle branch block
Source: Eur Heart J Imaging Methods Pract. 2026 May 14;4(1):qyag086. doi: 10.1093/ehjimp/qyag086 (PMC13222643; doi:10.1093/ehjimp/qyag086)
Supplement: qyag086_Supplementary_Data [file qyag086_supplementary_data.zip › Supplementary Material.docx]

**Supplementary data**

*Deformation imaging*

Our previously proposed 2-D HFR STE algorithm (Orlowska et al., 2020; Orlowska et al., 2021) is based on a two-step motion estimation using 1-D cross-correlation of 2-D kernels. Briefly, the axial displacement is determined from RF signals using 7°-wide, 4.5 mm-high kernels by analyzing consecutive frames. Then, lateral displacement is estimated from envelope-detected data using 16°-wide, 10 mm-high kernels, where a Hamming window is applied, and the mean pixel value is subtracted to enhance accuracy. Due to lower lateral resolution, frames are analyzed with a 30 ms time lag, compensating for axial shifts estimated in the first step. A 10:1 spline peak fitting ensures subpixel precision. For the contour tracking, an expert manually places reference points on the myocardium's mid-wall at end-diastole. A spline curve connects these points, forming a contour with ~2 mm spacing. To enhance robustness, eight supporting contours are automatically generated within a 4 mm band. A Savitzky-Golay filter smooths the motion to prevent unnatural movements. Finally, and for each apical view, the contour is then divided into six segments following EACVI/ASE guidelines. Segments labelled "non-tracked" are excluded from further analysis, while the remaining segments were used to calculate strain and strain rate. Strain is defined as the deformation of a contour and calculated as follows:

$S\left( t \right)= \frac{L\left( t \right)-L_{0}}{L_{0}}$ *(1)*

where L(t) is the length of the contour at time instance t and L_0_ is its initial length, i.e., at end-diastole (D’hooge et al., 2000). Furthermore, strain rate (SR), i.e., the speed at which deformation occurred, was estimated as:

$Sr\left( t \right)= \frac{\partial S(t)}{\partial t}$ *(2)*

**Correlation between electrical and mechanical activation**

A correlation, albeit moderate, was found between the duration of electrical and mechanical activation (r=0.593, p<0.001), as shown in Figure S7, whereas the QRS complex was consistently longer in duration compared to the duration of mechanical activation. However, the QRS complex represents both RV and LV depolarization, whereas we limited our analysis on the LV mechanical activation. Moreover, the QRS also represents transmural depolarization, whereas the contour used to track the myocardial movement was placed at the border of endo- to mid- myocardium, implying that the epicardial depolarization was not taken into account. Besides, studies using non-invasive imaging modalities, namely Cine DENSE MRI showed comparable mechanical activation times, both for HV, as well as for HF patients meeting the criteria for a CRT device implantation (24). On the other hand, it has been shown that electrical synchrony, and by extension a narrow QRS, might not be a prerequisite to achieving optimal and synchronous mechanical activation (27), which could explain why, even though there was no significant difference in the QRS duration between BiV off and BiV on, the duration of mechanical activation differed between the two states. Besides, in asynchronously activated hearts, such as in LBBB pattern of activation, the onset of shortening of later activated regions can also be delayed, given the high afterload imposed by the earlier activated regions that needs to be overcome before the myocardium starts to shorten. This further explains why there was a difference between the mechanical activation timings, while none was found between the electrical activation timings (QRS duration).

**Table S1. Original and alternative LV lead pacing pole for the 5 patients whose pacing settings were temporarily modified**

|  | Original pacing pole | Alternative pacing pole |
| --- | --- | --- |
| Patient #1 | LV tip | LV ring |
| Patient #2 | LV ring | LV tip |
| Patient #3 | LV3 | LV tip |
| Patient #4 | LV2 | LV4 |
| Patient #5 | LV1 | LV3 |

LV ring and LV4 are the most proximal to the device poles, followed by LV2 and LV3, whereas LV tip and LV1 are the most distal ones.

**Table S2. Onset and end of LV activation for each participant in the three study groups (HV, BiV off, BiV on)**

| No | b ant | b ant-sep | b inf-sep | b  inf | b inf-lat | b ant-lat | m ant-sep | m inf-sep | m inf-lat | m ant-lat | a ant | a sep | a inf | a lat | LV offset (ms) |
| --- | --- | --- | --- | --- | --- | --- | --- | --- | --- | --- | --- | --- | --- | --- | --- |
| HV1 |  | + |  |  | x |  |  |  |  |  |  |  | + |  |  |
| HV2 |  |  |  |  | x |  |  | + |  |  |  |  |  |  |  |
| HV3 |  |  |  |  | x |  | + |  |  |  |  |  |  |  |  |
| HV 4 |  |  |  |  | x |  | + |  |  |  |  |  |  |  |  |
| HV5 |  |  |  |  | x |  | + |  |  |  |  |  |  |  |  |
| HV6 |  |  |  |  | x |  | + |  |  |  |  |  |  |  |  |
| HV7 |  |  |  |  | x |  | + |  |  |  |  |  |  |  |  |
| HV8 |  |  | x |  |  |  | + |  |  |  |  |  |  |  |  |
| HV9 |  |  |  |  | x |  | + |  |  |  |  |  |  |  |  |
| HV10 |  |  |  |  | x |  |  | + |  |  |  |  |  |  |  |
| HV11 |  |  |  |  | x |  | + |  |  |  |  |  |  |  |  |
| HV12 |  |  |  |  | x |  | + |  |  |  |  |  |  |  |  |
| HV13 |  |  |  |  | x |  | + |  |  |  |  |  |  |  |  |
| HV14 |  |  |  |  | x |  | + |  |  |  |  |  |  |  |  |
| HV15 |  |  |  |  | x |  |  | + |  |  |  |  |  |  |  |
| HV16 |  |  |  |  | x |  | + |  |  |  |  |  |  |  |  |
| HV17 |  |  |  |  | x |  |  | + |  |  |  |  |  |  |  |
| HV18 |  |  |  |  | x |  | + |  |  |  |  |  |  |  |  |
| HV19 |  |  |  |  | x |  |  | + |  |  |  |  |  |  |  |
| HV20 |  |  |  |  | x |  | + |  |  |  |  |  |  |  |  |
| OFF1 |  |  |  |  | x |  | + |  |  |  |  |  |  |  |  |
| OFF2 |  |  |  |  |  | x |  | + |  |  |  |  |  |  |  |
| OFF3 |  |  |  |  |  |  | + |  |  | x |  |  |  |  |  |
| OFF4 |  |  |  |  | x |  | + |  |  |  |  |  |  |  |  |
| OFF5 |  |  |  |  |  |  |  | + |  | x |  |  |  |  |  |
| OFF6 |  |  |  |  |  |  | + |  |  | x |  |  |  |  |  |
| OFF7 |  |  |  |  |  |  |  |  |  | x |  | + |  |  |  |
| OFF8 |  |  |  |  |  |  |  | + | x |  |  |  |  |  |  |
| OFF9 |  |  |  |  |  |  |  | + |  | x |  |  |  |  |  |
| OFF10 |  |  |  |  | x |  | + |  |  |  |  |  |  |  |  |
| OFF11 |  |  |  |  |  | x |  | + |  |  |  |  |  |  |  |
| OFF12 |  |  |  |  | x |  | + |  |  |  |  |  |  |  |  |
| OFF13 |  |  | + |  | x |  |  |  |  |  |  |  |  |  |  |
| OFF14 |  |  |  |  | x |  |  | + |  |  |  |  |  |  |  |
| OFF15 |  |  |  |  | x |  | + |  |  |  |  |  |  |  |  |
| OFF16 |  | + |  |  |  | x |  |  |  |  |  |  |  |  |  |
| OFF17 |  |  |  |  |  |  | + |  | x |  |  |  |  |  |  |
| OFF18 |  |  |  |  |  | x |  | + |  |  |  |  |  |  |  |
| OFF19 |  |  |  |  |  |  | + |  |  | x |  |  |  |  |  |
| OFF20 |  |  |  |  |  | x |  |  |  |  |  | + |  |  |  |
| ON1 |  | **+_1_** |  |  | +_2_ |  |  |  | **++_2_** |  |  | ++_1_ |  |  | 20 |
| ON2 |  |  |  |  |  |  | **+_1_,++_1_** |  | +_2_,++_2_ |  |  |  |  |  | 20 |
| ON3 |  |  | +_1_ |  |  |  |  | ++_1_ |  | **+_2_,++_2_** |  |  |  |  | 20 |
| ON4 |  |  |  |  |  |  |  | +_1,_++_1_ | ++_2_ | **+_2_** |  |  |  |  | N/A |
| ON5 |  |  | **+_1_** |  | +_2_ |  |  | ++_1_ |  | ++_2_ |  |  |  |  | N/A |
| ON6 |  |  | **+_1_** |  |  |  | ++_1_ |  | +_2_,++_2_ |  |  |  |  |  | N/A |
| ON7 |  |  |  |  | +_2_ |  | **+_1,_++_1_** |  | ++_2_ |  |  |  |  |  | 40 |
| ON8 |  |  |  |  |  |  |  | +_1,_++_1_ | ++_2_ | **+_2_** |  |  |  |  | **N/A** |
| ON9 |  |  |  |  | +_2_ |  | **+_1_**_,_++_1_ |  | ++_2_ |  |  |  |  |  | N/A |
| ON10 |  |  |  |  | +_2_ |  | **+_1_**_,_++_1_ |  | **++_2_** |  |  |  |  |  | 40 |
| ON11 |  |  |  |  |  |  | +_1,_++_1_ |  | **+_2_,++_2_** |  |  |  |  |  | 30 |
| ON12 |  |  |  |  |  |  |  |  |  |  |  |  |  | **+_2_,++_2_** | LV paced |
| ON13 |  |  |  |  |  | ++_2_ | +_1,_++_1_ |  |  | **+_2_** |  |  |  |  | N/A |
| ON14 |  |  |  |  |  |  | +_1,_++_1_ |  |  |  |  |  | **+_2_** | ++_2_ | N/A |
| ON15 |  |  |  |  | +_2_,++_2_ |  | **+_1_** |  |  |  |  | ++_1_ |  |  | **N/A** |
| ON16 |  |  |  |  |  |  |  | +_1,_++_1_ |  | **+_2_**,++_2_ |  |  |  |  | N/A |
| ON17 |  |  |  |  |  |  |  | **+_1_**,++_1_ |  | +_2_,++_2_ |  |  |  |  | N/A |
| ON18 |  |  |  |  |  |  | ++_1_ | **+_1_** |  | +_2_,++_2_ |  |  |  |  | 30 |
| ON19 |  |  |  |  |  |  |  | **+_1,_++_1_** |  |  |  |  |  | +_2_,++_2_ | N/A |
| ON20 |  |  |  |  | **+_2_,++_2_** |  | ++_1_ |  |  |  |  | +_1_ |  |  | 30 |

b: basal, m: mid, a: apical, ant: anterior, sep: septal, inf: inferior, lat: lateral, OFF: biventricular pacing (BiV) off – LBBB pattern of activation, ON: BiV on, (+) earliest and (x) latest activated segment, +_1_ and +_2_ earliest activated segments during BiV on, based on HFR STE, for RV and LV lead respectively, (++) earliest activated segment based on fluoroscopic images during BiV on. LV offset is mentioned in case LV pacing lead was activated before the RV one. In bold the first activated segment during BiV on, as defined with HFR STE (**+**) and by fluoroscopy (**++**), if applicable.

**Table S3. Paired t-test of each segment of the 5 HV that were re-scanned and used for our method reproducibility assessment**

| Myocardial segment | p-value |
| --- | --- |
| Basal anterior | 0.372 |
| Basal anteroseptal | 0.777 |
| Basal inferoseptal | 0.439 |
| Basal inferior | 0.558 |
| Basal inferolateral | 0.526 |
| Basal anterolateral | 0.857 |
| Mid anterior | 0.556 |
| Mid anteroseptal | 0.250 |
| Mid inferoseptal | 0.335 |
| Mid inferior | 0.206 |
| Mid inferolateral | 0.799 |
| Mid anterolateral | 0.793 |
| Apical anterior | 0.967 |
| Apical septal | 0.249 |
| Apical inferior | 0.665 |
| Apical lateral | 0.385 |
| All segments | 0.909 |

**Table S4. Echocardiographic data of the 5 HV that were re-scanned and used for our method reproducibility assessment**

|  | **First scan** | **Second scan** | **p-value** |
| --- | --- | --- | --- |
| SBP (mmHg) | 117±12 | 117±9.8 | N/S |
| DBP (mmHg) | 60±6.1 | 59±4.2 | N/S |
| EDV (ml) | 110.6±24.1 | 116.8±24.8 | N/S |
| ESV (ml) | 46.2±11.7 | 47.6±14.3 | N/S |
| E (m/sec) | 0.78±0.57 | 0.82±0.7 | N/S |
| A (m/sec) | 0.48±0.11 | 0.5±0.12 | N/S |
| IVC (mm) | 18±2.6 | 18.2±2.8 | N/S |

Abbreviations as in Table 1. Data are expressed as mean ± SD

**Figure S1.** Examples of bull’s eye plots for two patients during BiV. The stars in both cases represent the position of the pacing poles of the right and left pacemaker lead. In (A), the position for both RV and LV was correctly identified (absolute difference in segments = 0 for both RV and LV lead), whereas in (B), the position of the RV lead was at the mid-anteroseptum and of the LV lead at the mid-inferolateral wall, but the measured earliest activated segment were basal inferoseptal (absolute difference in segments=2) and basal inferolateral (absolute difference in segments=1), respectively. Abbreviations as in Figure 1.

**Figure S2.** Examples of SR curves where the automatic zero-crossing identification failed. A to D: A first zero-crossing occurs before true zero-crossing reflecting the onset of isovolumic contraction (IVC), which can be attributed to noise. E: multiple zero crossings before IVC. F: A zero-crossing exists; however, it is not followed by a significant trough, which can be attributed to failure of HFR-STE to track the whole myocardial movement/deformation.

**Figure S3.** Bull’s eye showing the average activation pattern for all HV (A), BiV off (B) and BiV on (C).

**Figure S4.** Scatter diagram used to plot the relationship between the activation timings of the septal and lateral wall for the different groups. Spearman’s Rank correlation coefficient showed a moderate positive correlation (r = 0.424, p < 0.001). The blue line represent the linear regression line. The ratio of septal to lateral wall time of activation for the tree groups was: HV: 31.2/50=0.624, CRT OFF= 36.3/81.6=0.445, CRT ON= 55.57/54.54= 1.019 Abbreviations as in Figure 1.

**Figure S5.** Bland-Altman plot for the two measurements performed on each myocardial segment for the five HV. The bias is shown by the green solid line, and the limits of agreement  (1.95 × SD) by the dashed red lines.

**Figure S6.** Bland-Altman plots for the intra- (left) and inter- (right) observer variability. The bias is shown by the green solid line, and the limits of agreement  (1.95 × SD) by the dashed red lines.

**Figure S7.** Mechanical activation duration plotted as a function of electrical activation duration for the different groups, showing a moderate positive correlation (r =0.593, p<0.001). Abbreviations as in Central Illustration, BiV on diff set: biventricular pacing but after changing the pacing pole of the LV lead.

**Figure S8.** Corresponding 12lead ECGs of the activation maps shown on Figure 2 (from left to right: HV, LBBB, CRT on).
