# Supplementary figures and images for: High frame rate speckle tracking echocardiography to image the left ventricular mechanical activation sequence in healthy participants and patients with left bundle branch block
Source: Eur Heart J Imaging Methods Pract. 2026 May 14;4(1):qyag086. doi: 10.1093/ehjimp/qyag086 (PMC13222643; doi:10.1093/ehjimp/qyag086)

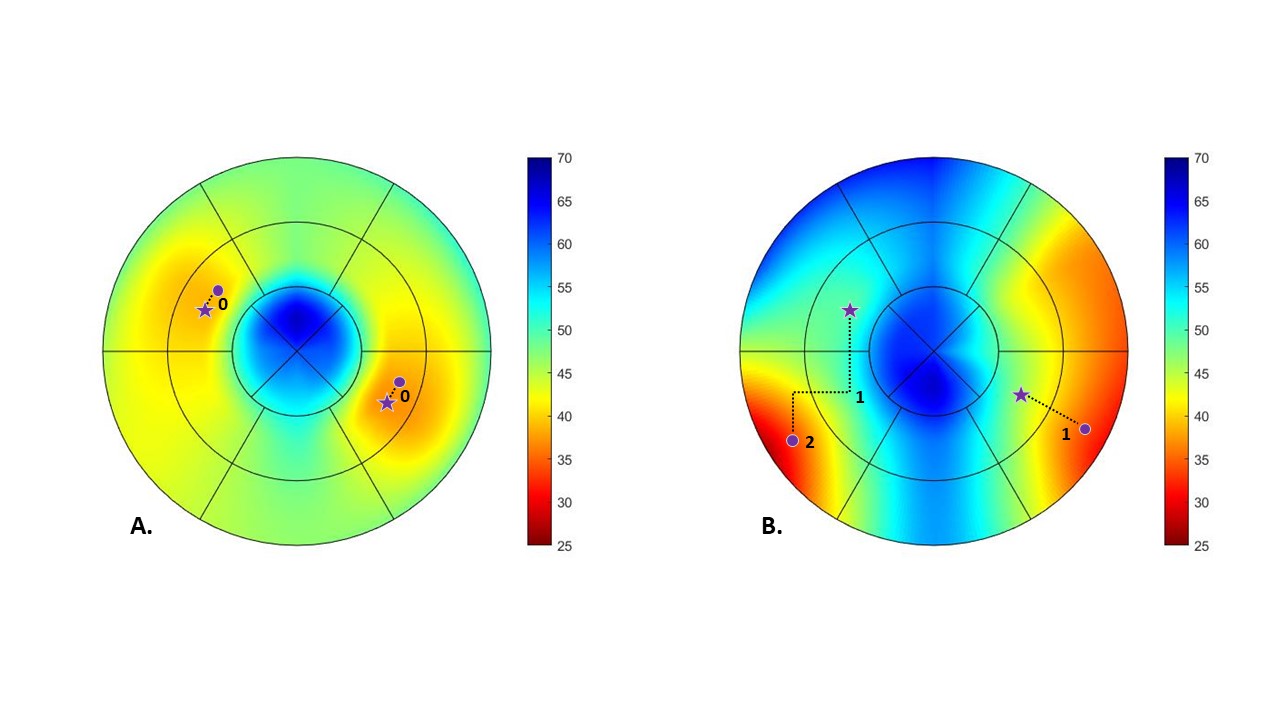

Supplement: qyag086_Supplementary_Data [file qyag086_supplementary_data.zip › Figure S1.jpg]

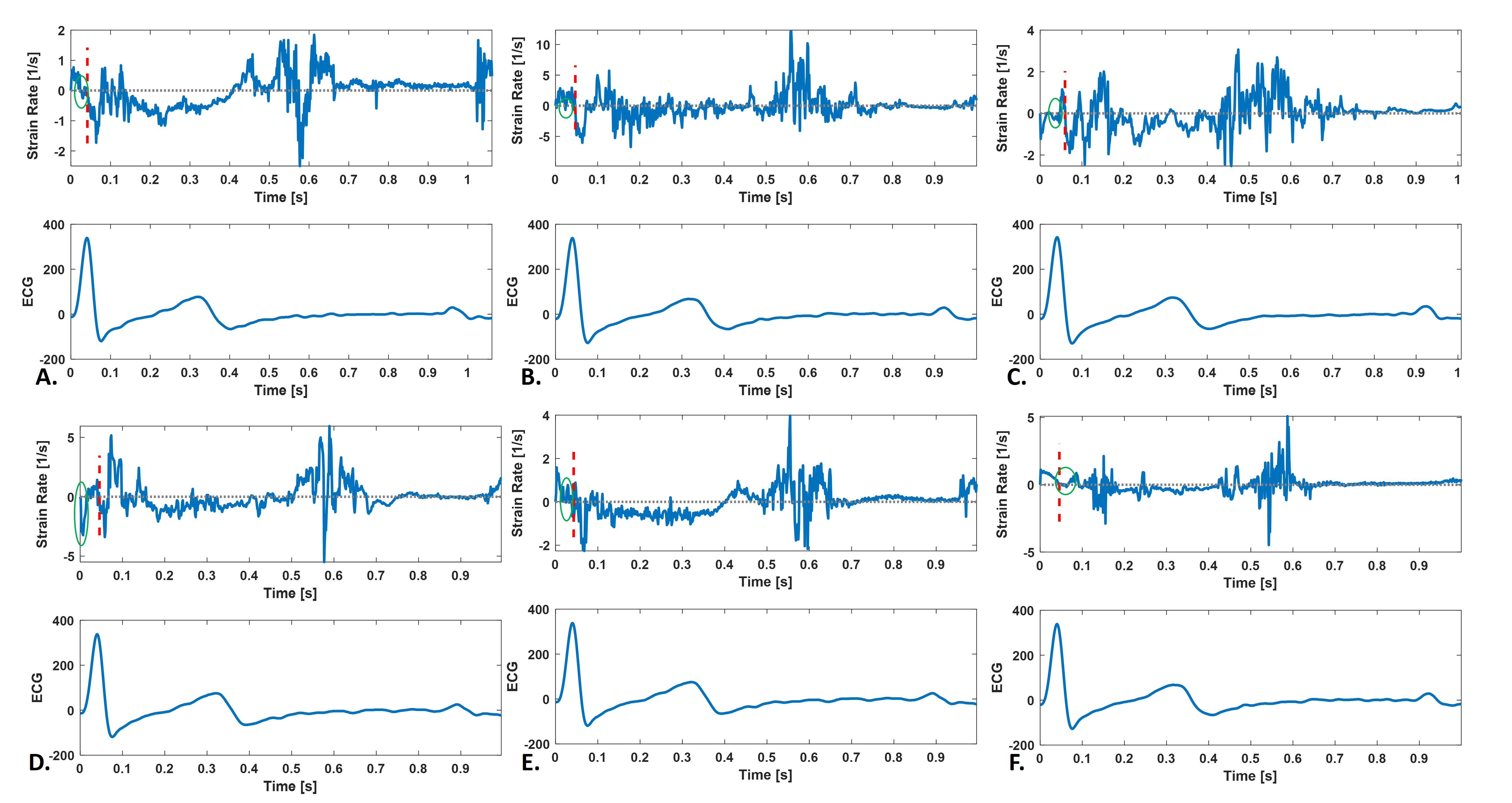

Supplement: qyag086_Supplementary_Data [file qyag086_supplementary_data.zip › Figure S2.jpg]

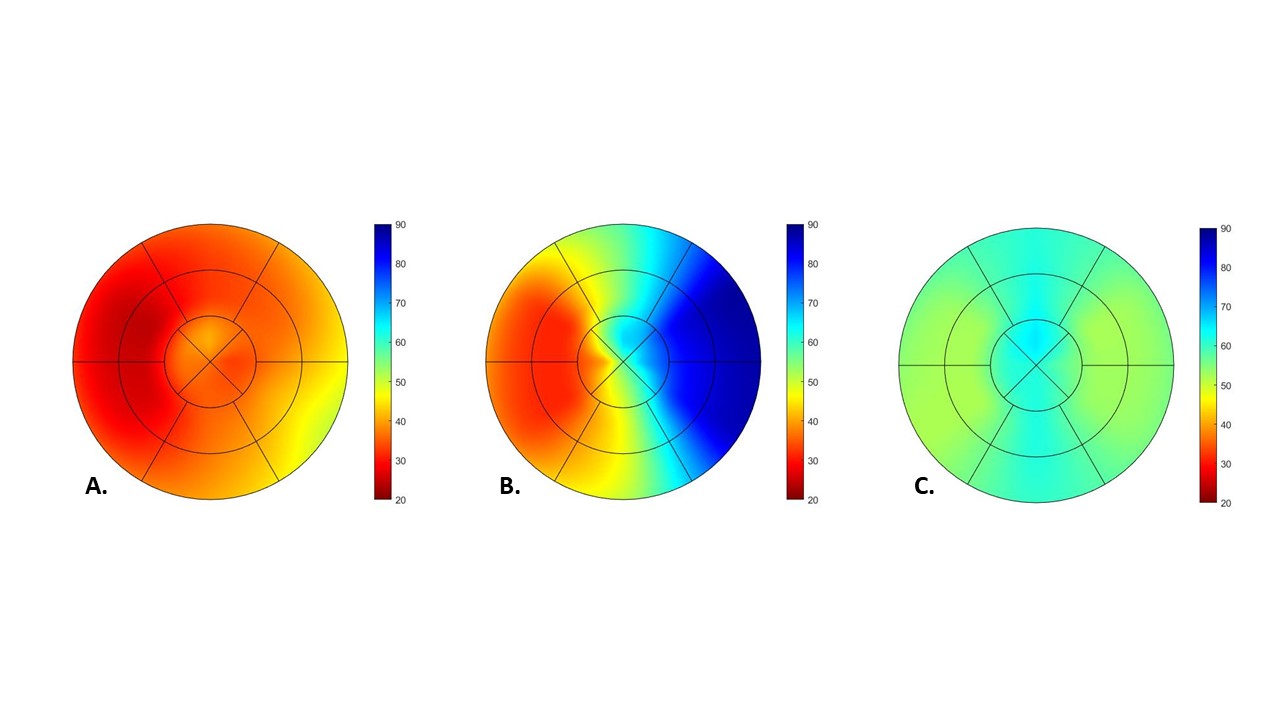

Supplement: qyag086_Supplementary_Data [file qyag086_supplementary_data.zip › Figure S3.jpg]

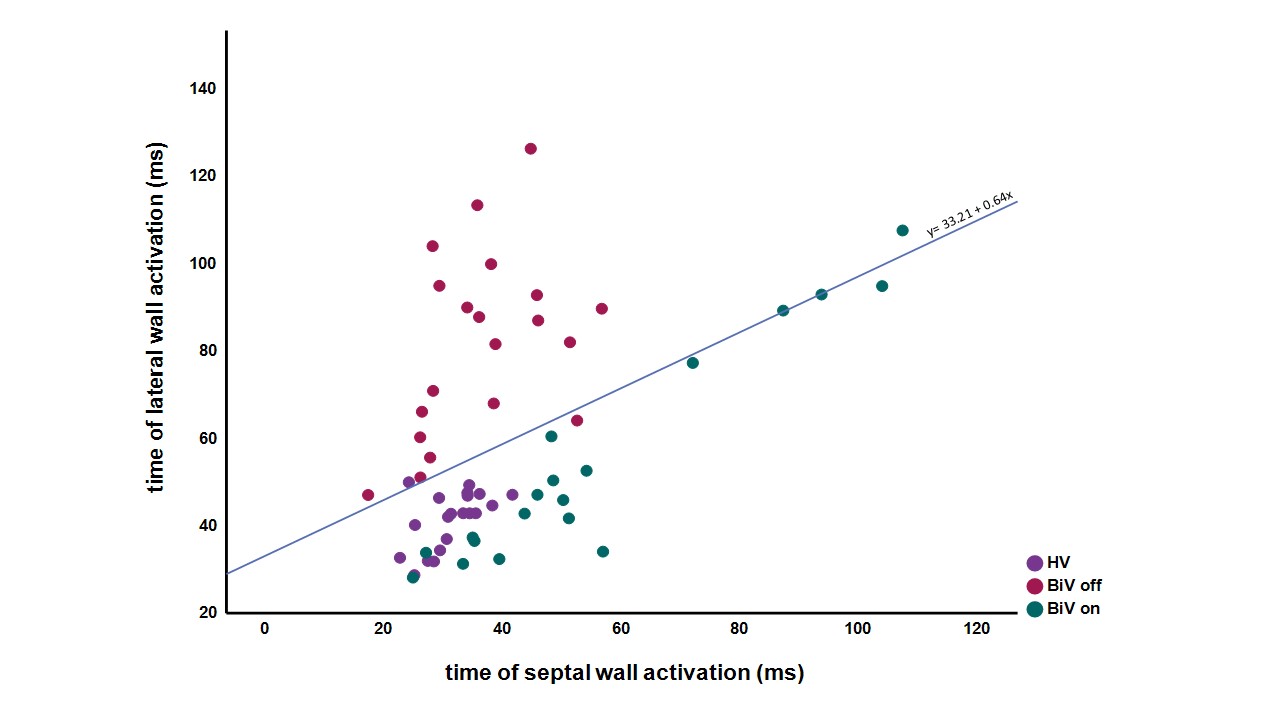

Supplement: qyag086_Supplementary_Data [file qyag086_supplementary_data.zip › Figure S4.jpg]

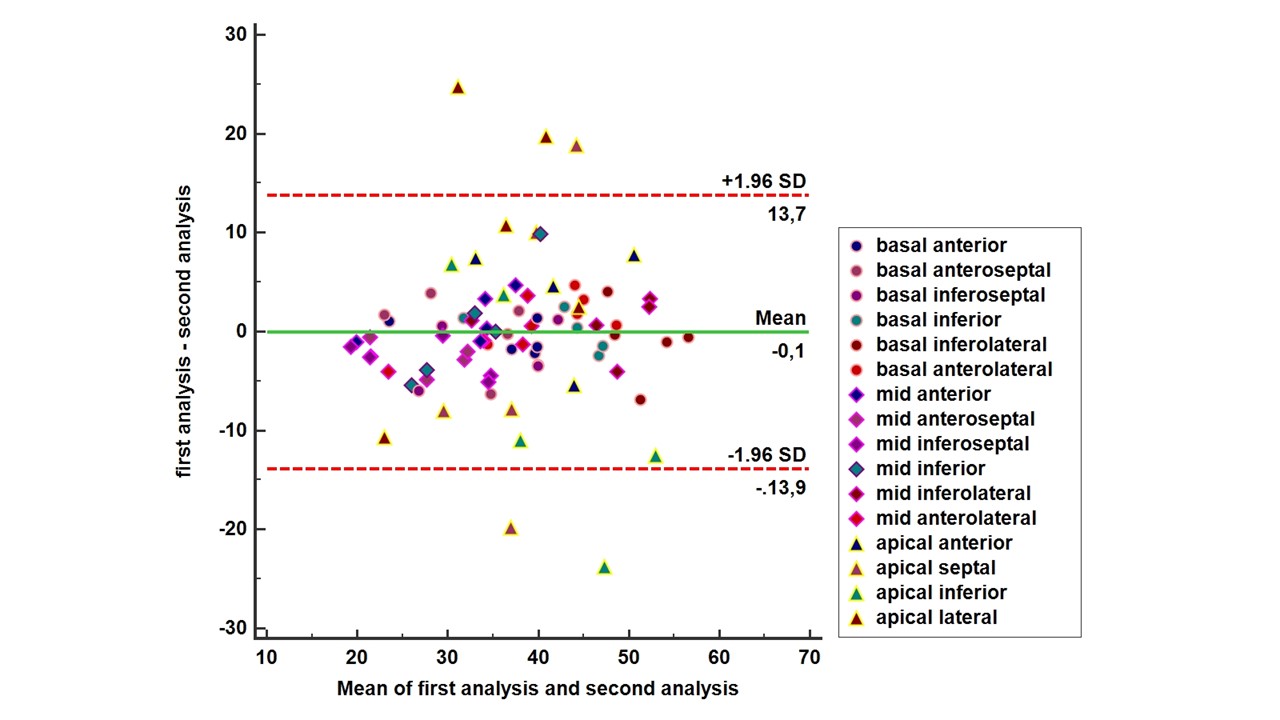

Supplement: qyag086_Supplementary_Data [file qyag086_supplementary_data.zip › Figure S5.jpg]

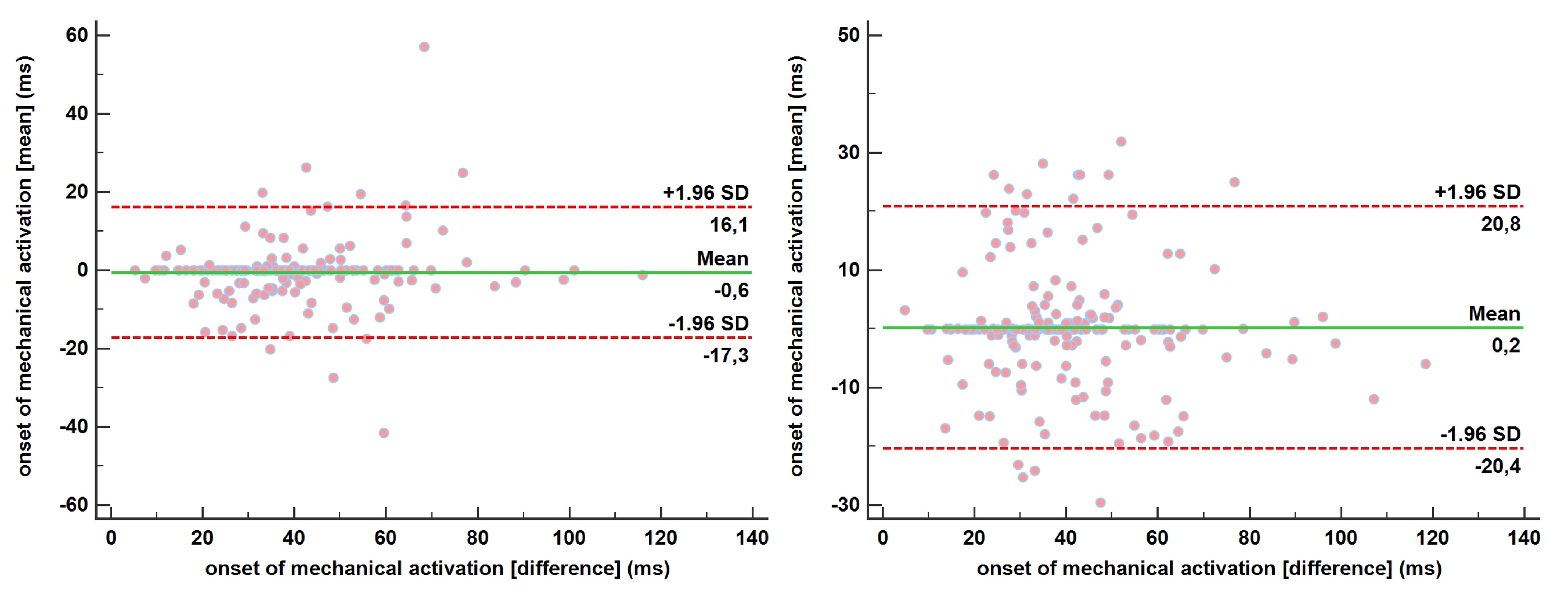

Supplement: qyag086_Supplementary_Data [file qyag086_supplementary_data.zip › Figure S6.jpg]

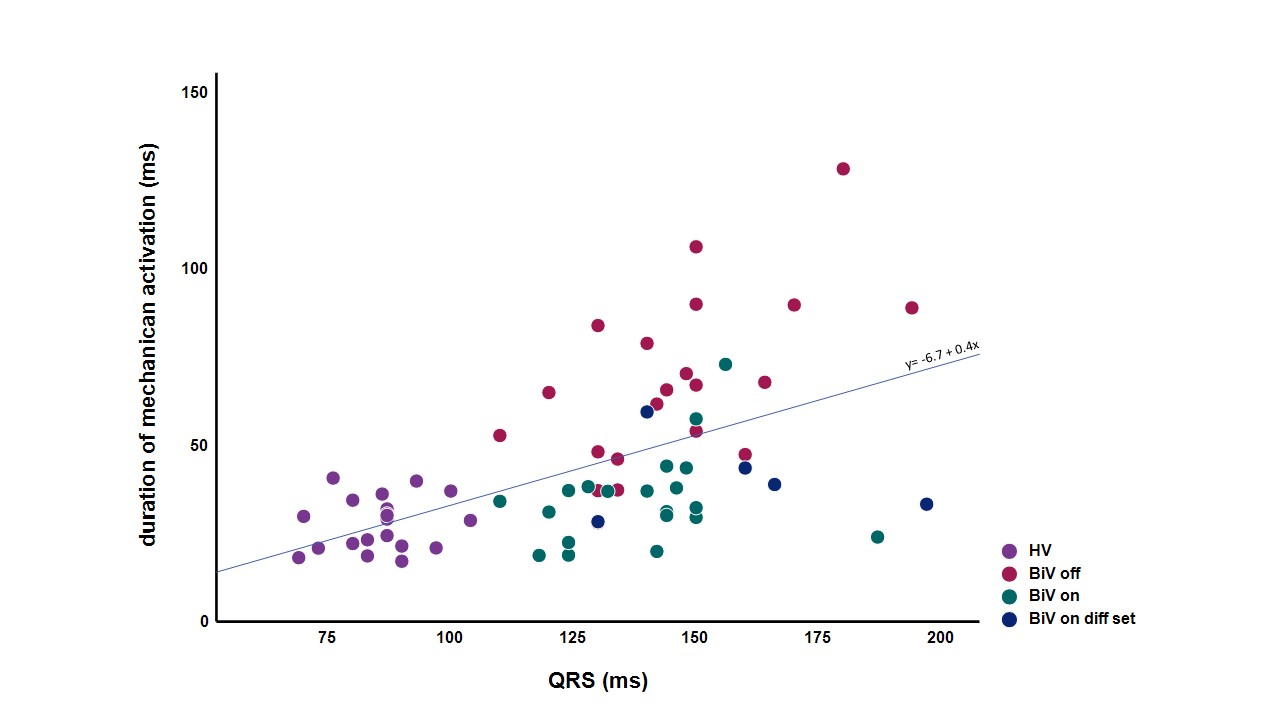

Supplement: qyag086_Supplementary_Data [file qyag086_supplementary_data.zip › Figure S7.jpg]

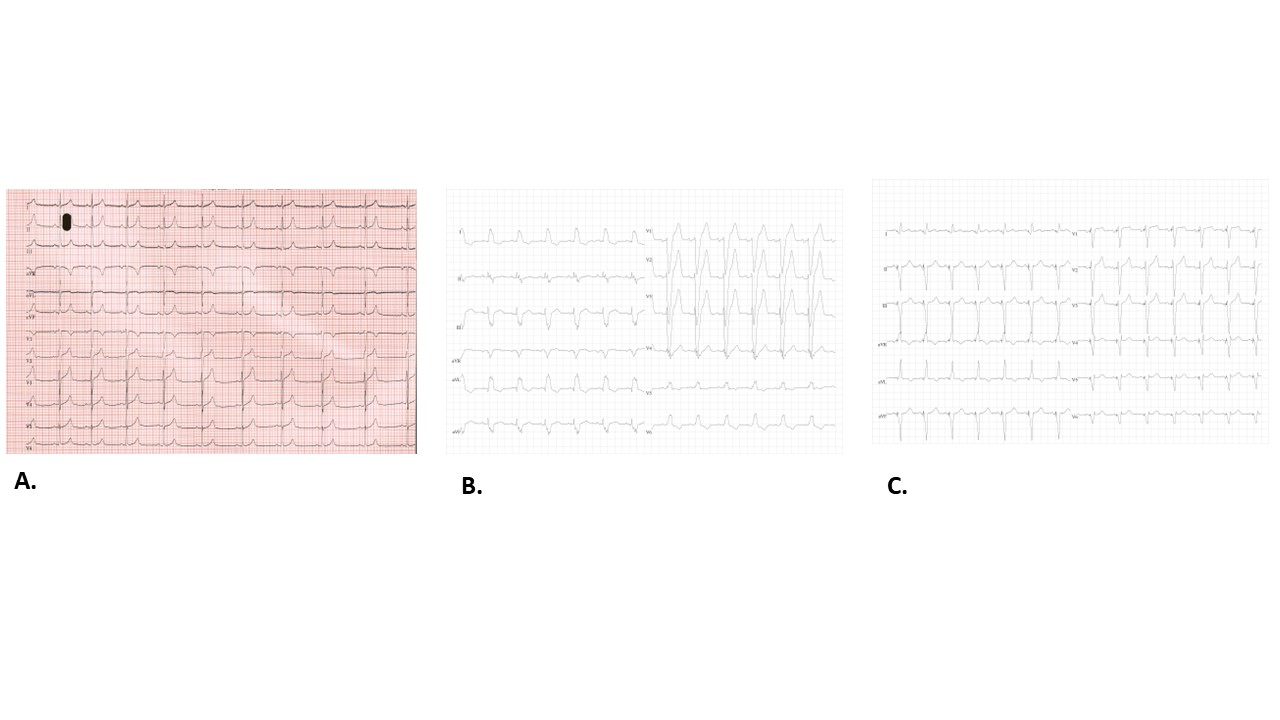

Supplement: qyag086_Supplementary_Data [file qyag086_supplementary_data.zip › Figure S8.jpg]
